# Validation of the Southend giant cell arteritis probability score in a Scottish single-centre fast-track pathway

**DOI:** 10.1093/rap/rkab102

**Published:** 2021-12-15

**Authors:** Andrew R Melville, Karen Donaldson, James Dale, Anna Ciechomska

**Affiliations:** 1 Institute of Infection, Immunity & Inflammation, University of Glasgow, Glasgow; 2 Rheumatology Department, University Hospital Wishaw, NHS Lanarkshire; 3 School of Medicine, Dentistry and Nursing, University of Glasgow, Glasgow, UK

**Keywords:** GCA, temporal arteritis, probability score, fast-track pathway, ultrasound, diagnosis

## Abstract

**Objective:**

The aim was to provide external validation of the Southend GCA probability score (GCAPS) in patients attending a GCA fast-track pathway (GCA FTP) in NHS Lanarkshire.

**Methods:**

Consecutive GCA FTP patients between November 2018 and December 2020 underwent GCAPS assessment as part of routine care. GCA diagnoses were supported by US of the cranial and axillary arteries (USS), with or without temporal artery biopsy (TAB), and confirmed at 6 months. Percentages of patients with GCA according to GCAPS risk group, performance of total GCAPS in distinguishing GCA/non-GCA final diagnoses, and test characteristics using different GCAPS binary cut-offs were assessed. Associations between individual GCAPS components and GCA and the value of USS and TAB in the diagnostic process were also explored.

**Results:**

Forty-four of 129 patients were diagnosed with GCA, including 0 of 41 GCAPS low-risk patients (GCAPS <9), 3 of 40 medium-risk patients (GCAPS 9–12) and 41 of 48 high-risk patients (GCAPS >12). Overall performance of GCAPS in distinguishing GCA/non-GCA was excellent [area under the receiver operating characteristic curve, 0.976 (95% CI 0.954, 0.999)]. GCAPS cut-off ≥10 had 100.0% sensitivity and 67.1% specificity for GCA. GCAPS cut-off ≥13 had the highest accuracy (91.5%), with 93.2% sensitivity and 90.6% specificity. Several individual GCAPS components were associated with GCA. Sensitivity of USS increased by ascending GCAPS risk group (nil, 33.3% and 90.2%, respectively). TAB was diagnostically useful in cases where USS was inconclusive.

**Conclusion:**

This is the first published study to describe application of GCAPS outside the specialist centre where it was developed. Performance of GCAPS as a risk stratification tool was excellent. GCAPS might have additional value for screening GCA FTP referrals and guiding empirical glucocorticoid treatment.

Key messages

- External validation confirms the value of the Southend GCA probability score as a risk assessment and also diagnostic tool.
- A GCA probability score <10 may be sufficient to exclude GCA without fast-track review.
- Patients with a GCA probability score ≥13 appear high risk for GCA and warrant empirical glucocorticoids.

## Introduction

International guidelines recommend temporal and axillary artery US as a first-line modality for confirmation of diagnoses of GCA [1, 2]. Fast-track GCA pathways (GCA FTP) with application of US are growing in popularity; however, the number and availability of trained sonographers is a potential limiting factor. Delayed clinical assessment carries the risk of complications, including loss of vision [3], in addition to jeopardizing the value of clinical findings and test results in patients started on glucocorticoids by their referring clinician.

Risk stratification scores could help prioritize referrals to GCA FTPs, facilitating timely assessment and treatment of patients with GCA, in addition to avoidance of over-treatment with glucocorticoids in those with other diagnoses. The Southend GCA probability score (GCAPS), also known as the Southend GCA clinical pre-test probability score [4], developed in a specialist centre at Southend University Hospital, has shown promise in discriminating patients with low, medium and high pre-test probability for GCA [5] and appears to augment the diagnostic performance of US. There is a suggestion that GCAPS alone might be sufficient to exclude GCA in low-risk patients, without additional tests, which, if confirmed, could have major significance for resource allocation. These findings require validation, particularly given the potentially serious consequences of missed diagnoses. Evidence to support the use of GCAPS in external cohorts is currently limited, consisting of conference reports but no published studies [6, 7].

National Health Service (NHS) Lanarkshire is a health board in Scotland (UK) serving a population of 650 000 people across urban and rural communities. A GCA FTP was established in 2018, based on the earlier experiences of FTPs in other parts of the UK [7, 8].

The aim of this study was to assess the performance of GCAPS in our cohort of patients from NHS Lanarkshire, including the ability of GCAPS to categorize patients at the pre-assessment stage to groups of low, medium and high risk, the ability of GCAPS to discriminate GCA from non-GCA final diagnoses, and whether GCAPS alone might be sufficient to exclude GCA in patients deemed low risk (i.e. below a specified binary cut-off).

Additional aims were to explore the predictive values of individual GCAPS components in this dataset, to report the diagnostic yield of US of the cranial and axillary arteries (USS) across GCAPS risk groups and to describe the role of temporal artery biopsy (TAB) in the diagnostic process.

## Methods

### Study design

We conducted a retrospective cohort study of consecutive patients with suspected GCA assessed on the NHS Lanarkshire GCA FTP over a 2-year period (from November 2018 to December 2020). According to NHS Research Ethics Committee guidelines, this work was classed as service evaluation, and formal ethical approval was not required [9].

### Clinical practice

The NHS Lanarkshire GCA FTP was supported by two consultant rheumatologists. Referral sources included primary and secondary care. Approximately 30% of referrals were rejected following telephone consultation on grounds of clinical implausibility (e.g. combination of young age, normal inflammatory indicators and clear alternative diagnosis). The remaining patients were assessed in person.

GCAPS evaluation was incorporated into routine clinical practice as an assessment aid and promising pre-test tool to prioritize referrals. All patients underwent USS, with additional tests (e.g. TAB, cross-sectional imaging) arranged at the discretion of the treating consultant. Diagnostic and therapeutic decisions were made on a case-by-case basis by the treating consultant.

US scans were performed by trained sonographers (A.C. and K.D.) at the time of initial assessment, with GE Logiq S8 (linear probe ML6-15) and GE Logiq e (linear probe L4-12t) scanners, using settings recommended by EULAR [1]. Non-compressible halo sign and intima media thickness (IMT) were recorded for the superficial temporal artery, frontal and parietal temporal artery branches and facial artery, in both longitudinal and transverse planes. IMT was recorded at the carotid, subclavian and axillary arteries.

### Definitions

For this study, GCA diagnosis was defined pragmatically by the decision of the clinician to treat as GCA after FTP review, with confirmation 6 months after the initial visit. Most cases were supported by additional tests showing objective evidence of cranial and/or large vessel vasculitis, according to BSR and EULAR recommendations [1, 2]. In some cases, additional test results were negative or inconclusive, but mitigating circumstances existed to explain non-positive results. Non-GCA diagnoses were also confirmed after 6 months by review of clinical notes.

Positive USS was defined by the presence of a non-compressible halo sign (according to OMERACT definitions [10]), associated with decreased echogenicity of cranial arterial walls, and maximal IMT measurement ≥0.5 mm for the temporal artery, ≥0.4 mm for the frontal and parietal temporal artery branches and the facial artery, and >1 mm for subclavian and axillary arteries [11, 12]. US scans demonstrating short sections of modestly thickened and hyperechoic IMT, irregular arterial walls (suggestive of atheromatic plaques) or borderline measurements were considered inconclusive [10].

GCAPS is a pre-test probability scoring system composed of positive and negative integers based on patient demographics, symptoms, signs, laboratory findings and competing differentials encountered during assessment of suspected GCA. Previous publications have considered scores of <9 to be low risk for GCA, 9–12 medium risk and >12 high risk [4, 5].

### Data collection

Patient demographics, time from referral to assessment, duration of CS treatment, fulfilment of ACR classification criteria for GCA (excluding TAB information) [13], clinical features at presentation, including GCAPS components (symptom duration, CRP, cranial pain, constitutional symptoms, PMR, ischaemic symptoms, visual abnormalities, temporal artery abnormalities, extra-cranial vascular abnormalities, cranial nerve palsies, alternative diagnosis more likely], total score, results of USS, with or without TAB, and final diagnosis were collected routinely at the first visit and follow-up visits and recorded in a local clinical database, anonymized before analyses.

### Data analysis

Descriptive characteristics were expressed as the number (*n*) (%), mean (S.d.) or median [interquartile range (IQR)], depending on the data type and distribution. The performance of GCAPS in predicting a final diagnosis of GCA was assessed by receiver operating characteristic (ROC) analyses, and the sensitivity, specificity, positive predictive value, negative predictive value and accuracy of different GCAPS binary cut-off values were calculated. Multivariable logistic regression was used to test the associations between individual GCAPS components (positive integers only) and confirmed GCA. The sensitivity, specificity, positive predictive value, negative predictive value and accuracy of USS for GCA diagnosis, in the whole group and according to GCAPS risk group, were also calculated.

Patients with ineligible or insufficient data were excluded from analyses. Minimal missing data were anticipated, and no imputation of missing data was planned. Analyses were carried out using IBM SPSS Statistics v.21.0 (IBM) and R v.4.1.2 (R Core Team).

## Results

### Clinical characteristics

One hundred and thirty-three patients were assessed on the GCA FTP during the study period, of whom 129 had evaluable data and were included in analyses (of 4 excluded, 1 was referred with an existing GCA diagnosis, 1 was referred after an incidental finding of vasculitis on PET-CT, and 2 were lost to follow-up before confirmation of diagnosis).

Eighty-three of 129 (64.3%) were female, and their mean (S.d.) age was 69.6 (9.5) years. The median (IQR) time from referral to assessment was 3 (2, 5) days. Ninety-three (72.1%) patients had received glucocorticoids for ≥24 h at the time of assessment, with a median (IQR) time on CSs of 4 (1, 7) days.

In the whole group of 129 patients, GCAPS ranged from 2 to 24, with a median (IQR) score of 10 (8, 15). Forty-one (31.8%) patients were classed as low risk (GCAPS <9), 40 (31.0%) as medium risk (GCAPS 9–12) and 48 (37.2%) as high risk (GCAPS >12).

### Diagnostic pathway

**Fig. 1:**
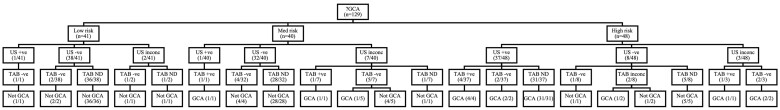
Flow chart showing route to diagnosis for all patients, according to GCA probability score risk group GCAPS: GCA probability score.

A confirmed diagnosis of GCA was made in 44 of 129 patients (34.1%). Of these, 33 (75.0%) fulfilled ACR classification criteria for GCA (excluding TAB information). Fig. 1 depicts the route to diagnosis for all patients, organized by GCAPS risk group. All 129 patients were assessed by USS, and 27 (20.9%) had TAB. In 40 of 44 cases, GCA diagnosis was supported by positive USS, positive TAB, or both. Of the remaining 4, 2 were diagnosed clinically, 1 developed scalp necrosis with a skin biopsy result suggestive of GCA, and 1 developed relapse after initial assessment following rapid CS taper, with subsequent USS positive (see Supplementary Table S1, available at *Rheumatology Advances in Practice* online).

**Fig. 2:**
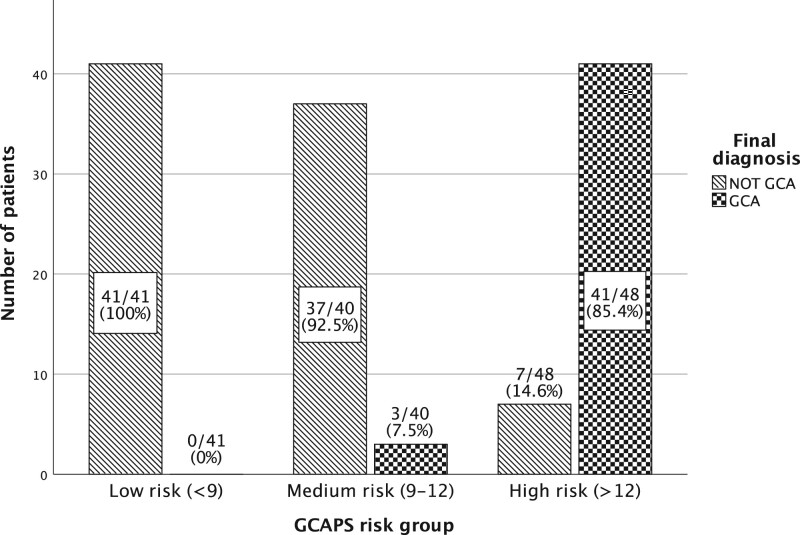
Bar chart displaying final GCA diagnosis by GCA probability score risk group GCAPS: GCA probability score.

Fig. 2 shows the final number of patients with GCA according to risk group. There were no patients with GCA in the low-risk group, 3 in the medium-risk group (7.5%) and 41 in the high-risk group (85.4%).

### GCAPS components

#### Overview

Table 1 shows GCAPS components and total scores for the whole group, by GCAPS risk group and by final GCA diagnosis. Most patients presented with cranial pain [120 of 129 (93.0%)], with similar percentages across risk groups and between GCA and non-GCA groups. Only 3 of 129 patients (2.3%) were <50 years of age, none of whom had GCA. No cases of GCA had CRP <10 mg/L. Ophthalmological abnormalities (i.e. anterior ischaemic optic neuropathy, central retinal artery occlusion, visual field defect or relative afferent pupillary defect) were rare, occurring in 7 of 129 patients (5.4%; 2 of 7 medium risk, 5 of 7 high risk; 5 of 7 GCA, 2 of 7 non-GCA).

**Table 1:** GCA probability score total score and selected components for all patients, by risk group and by final GCA diagnosis

Parameter	All (*n* = 129)	Low risk (*n* = 41)	Med risk (*n* = 40)	High risk (*n* = 48)	GCA (*n* = 44)	Not GCA (*n* = 85)
GCAPS, median (IQR)	10 (8, 15)	6 (5.5, 7.5)	10 (9, 11)	16 (14, 18)	16 (14, 18)	9 (6, 11)
Age, *n* (%), years						
<50	3 (2.3)	2 (4.9)	1 (2.5)	0 (0)	0 (0)	3 (3.5)
50–60	19 (14.7)	5 (12.2)	9 (22.5)	5 (10.4)	3 (6.8)	16 (18.8)
61–65	21 (16.3)	13 (31.7)	1 (2.5)	7 (14.6)	6 (13.6)	15 (17.6)
>65	86 (66.7)	21 (51.2)	29 (72.5)	36 (75.0)	35 (79.5)	51 (60.0)
Female, *n* (%)	83 (64.3)	22 (53.7)	31 (77.5)	30 (62.5)	24 (54.5)	59 (69.4)
CRP, mg/L^a^, *n* (%)						
0–5	30 (23.4)	19 (46.3)	11 (27.5)	0 (0)	0 (0)	30 (35.3)
6–10	8 (6.3)	4 (9.8)	3 (7.5)	1 (2.1)	0 (0)	8 (9.4)
11–25	22 (17.2)	11 (26.8)	10 (25.0)	1 (2.1)	2 (4.7)	20 (23.5)
>25	68 (53.1)	7 (17.1)	16 (40.0)	45 (95.7)	41 (95.3)	27 (31.8)
Cranial pain, *n* (%)	120 (93.0)	38 (92.7)	36 (90.0)	46 (95.8)	42 (95.5)	78 (91.8)
Constitutional^b^, *n* (%)						
Single	35 (27.1)	5 (12.2)	11 (27.5)	19 (39.6)	16 (36.4)	19 (22.4)
Combined	19 (14.7)	1 (2.4)	4 (10.0)	14 (29.2)	14 (31.8)	5 (5.9)
PMR	27 (20.9)	1 (2.4)	10 (25.0)	16 (33.3)	16 (36.4)	11 (12.9)
Ischaemic^c^	45 (34.9)	3 (7.3)	9 (22.5)	33 (68.8)	30 (68.2)	15 (17.6)
Visual^d^	7 (5.4)	0 0	2 (5.0)	5 (10.4)	5 (11.4)	2 (2.4)
Temporal artery, *n* (%)						
Tender	24 (18.6)	7 (17.1)	4 (10.0)	13 (27.1)	12 (27.3)	12 (14.1)
Thickened	10 (7.8)	0 (0)	1 (2.5)	9 (18.8)	9 (20.5)	1 (1.2)
Pulseless	6 (4.7)	0 (0)	0 (0)	6 (12.5)	6 (13.6)	0 (0)

a One missing CRP value (high-risk/GCA groups).

b Constitutional symptoms: night sweats, weight loss and fever.

c Ischaemic symptoms: jaw/tongue claudication, uniocular blurring, diplopia and amaurosis fugax.

d Ophthalmological abnormality: anterior ischaemic optic neuropathy, central retinal artery occlusion, visual field defect or relative afferent pupillary defect.

GCAPS: GCA probability score; IQR: interquartile range; PMR: symptoms suggestive of PMR.

Supplementary Table S2, available at *Rheumatology Advances in Practice* online, shows additional GCAPS data for the whole group and subgroups as in Table 1. Extracranial vascular signs (i.e. bruits or loss of pulse) and cranial nerve palsies were rare, affecting 5 of 129 (3.9%) and 2 of 129 (1.6%), respectively. Alternative diagnosis more likely than GCA occurred in 57 of 129 patients (44.2%).

#### Regression analyses

In multivariable logistic regression analyses, the following GCAPS components were associated with final diagnosis of GCA: increasing age group [odds ratio (OR) 4.59 (95% CI 1.17, 17.95); *P* = 0.03], increasing CRP [OR 7.45 (95% CI 1.80, 30.88); *P* = 0.006], presence of combined constitutional symptoms [compared with absence of constitutional symptoms; OR 34.72 (95% CI 2.45, 491.55); *P* = 0.009], presence of ischaemic symptoms [OR 29.96 (95% CI 3.43, 262.07); *P* = 0.002], presence of temporal artery tenderness [OR 16.19 (95% CI 1.63, 160.51); *P* = 0.02] and presence of temporal artery thickening [OR 92.98 (95% CI 4.59, 1871.49); *P* = 0.003] (both compared with absence of temporal artery changes). Female sex was associated with reduced likelihood of GCA [OR 0.08 (95% CI 0.01, 0.66); *P* = 0.02] (see Supplementary Table S3, available at *Rheumatology Advances in Practice* online).

#### Reciver operating characteristic curve analyses

**Fig. 3:**
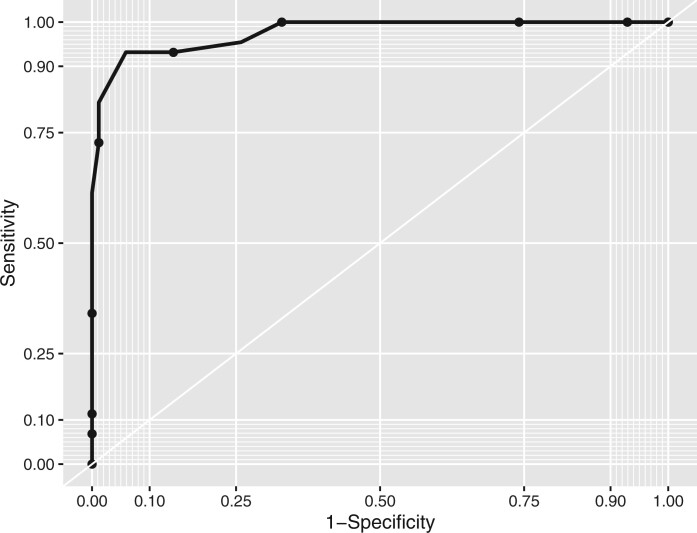
Receiver operating characteristic curve for total GCA probability score in predicting final diagnosis of GCA Area under the curve = 0.976 (95% CI 0.954, 0.999). Maximum Youden index (sensitivity + specificity−1) = 0.873, corresponding to a GCAPS binary cut-off value of ≥13. GCAPS: GCA probability score; ND: not done; TAB: temporal artery biopsy.

Fig. 3 shows the ROC curve for overall performance of GCAPS in discriminating final diagnoses of GCA from non-GCA, with an area under the curve of 0.976 (95% CI 0.954, 0.999), indicating excellent diagnostic ability [14]. The maximum Youden index (a measure of optimal compromise between sensitivity and specificity) [15] was 0.873, corresponding to a GCAPS binary cut-off value of ≥13.

### Binary cut-offs

Using a GCAPS binary cut-off of ≥9, 44 of 88 patients were correctly identified as GCA and 41 of 41 as non-GCA, giving a sensitivity of 100.0% and specificity of 48.2%. Using a cut-off of ≥10, sensitivity remained 100.0%, but specificity was higher, at 67.1%. Maximum accuracy (91.5%) was seen with a cut-off of ≥13 (also the maximum Youden index in ROC analyses). These results, along with additional cut-off values, are detailed in Table 2.

**Table 2:** Diagnostic performance of different GCA probability score binary cut-off values

GCAPS cut-off	Sensitivity (%)	Specificity (%)	Positive predictive value (%)	Negative predictive value (%)	Accuracy (%)
≥8^a^	100.0	36.5	44.9	100.0	58.1
≥9	100.0	48.2	50.0	100.0	65.9
≥10	100.0	67.1	61.1	100.0	78.3
≥11	95.5	74.1	65.6	96.9	81.4
≥12	93.2	83.5	74.5	95.9	86.8
≥13	93.2	90.6	83.7	96.3	91.5
≥14	79.5	97.6	94.6	90.2	91.5
≥15	72.7	98.8	97.0	87.5	89.9
≥16	61.4	100	100	83.3	86.8

a GCAPS cut-off of ≥8 means a score of ≥8 is considered test positive and a score of <8 test negative.

GCAPS: GCA probability score.

### US performance

In the whole group, 38 of 39 (97.4%) patients with a positive USS had a final diagnosis of GCA, and 84 of 90 patients (93.3%) with a negative or inconclusive USS had a non-GCA final diagnosis. Comparing positive scans with scans that were negative or inconclusive, USS had an overall sensitivity of 86.4% and specificity of 98.8%.

Sensitivity increased by risk group (incalculable for low-risk group because there was only 1 positive USS and no final diagnoses of GCA, 33.3% for medium risk, 90.2% for high risk). There were 2 of 41 (4.9%) inconclusive scans in the low-risk group, 7 of 40 (17.5%) in the medium-risk group and 3 of 48 (6.3%) in the high-risk group. Diagnostic performance of USS is summarized in Supplementary Table S4, available at *Rheumatology Advances in Practice* online.

Of patients with a final diagnosis of GCA and positive USS, 31 of 38 were positive for the temporal artery or temporal artery branches, 1 of 38 was positive for large vessels only, and 6 of 38 were positive for both the temporal artery (or branches) and large vessels. No cases were identified where a GCA diagnosis was based on facial, carotid or subclavian artery measurements alone (data available in 21 of 38).

Only one patient with a negative USS had a final diagnosis of GCA; this was a clinical diagnosis (TAB inconclusive; see Supplementary Table S2, available at *Rheumatology Advances in Practice* online). A further four patients with inconclusive USS had final GCA diagnoses. Conversely, only one patient with a positive USS had a final non-GCA diagnosis. This patient had pre-existing RA and developed symptoms suggestive of PMR, but had only partial response to moderate-dose glucocorticoids. GCAPS was 8. USS assessment of temporal arteries was positive unilaterally, with borderline axillary artery IMT changes. TAB (performed after 3 weeks on glucocorticoids) and PET-CT were negative. The patient was ultimately treated with tocilizumab for active RA, with resolution of symptoms, and repeat US was not undertaken.

### Temporal artery biopsy results

Of 27 TABs performed, 7 were positive, 18 negative and 2 inconclusive (see Fig. 1 for TAB results with corresponding GCAPS risk group, USS result and final diagnosis).

The TAB result was consistent with the USS result and final diagnosis in 12 cases; 5 had positive USS and positive TAB (all GCA); 7 had negative USS and negative TAB (all non-GCA). Conflicting USS and TAB results were seen in 3 cases; all had positive USS and negative TAB; 2 had a final diagnosis of GCA, 1 non-GCA (the patient described in the *USperformance* section). No patient had negative USS followed by positive TAB (of 9 performed). In 10 cases, TAB was performed after inconclusive USS; of these, the final diagnosis reflected the TAB result in 7 cases (2 diagnosed with GCA after positive TAB, 5 non-GCA after negative TAB), but in 3 cases a diagnosis of GCA was reached despite negative TAB (see Supplementary Table S2, available at *Rheumatology Advances in Practice* online). TAB was inconclusive in 2 cases, both after negative USS in GCAPS high-risk patients; 1 had a final diagnosis of GCA (see Supplementary Table S2, available at *Rheumatology Advances in Practice* online), 1 non-GCA.

## Discussion

This is the first published study of application of GCAPS outside the specialist centre where it was developed. In our setting, a newly established GCA FTP where GCAPS was incorporated as part of standard clinical assessment, the overall performance of GCAPS in predicting final GCA diagnoses was excellent. The prevalence of GCA increased across GCAPS risk groups, with none in the low-risk group, 7.5% in the medium-risk group and 85.4% in the high-risk group, indicating effective stratification at the pre-assessment stage. These results are comparable to previous publications from Southend [4, 5] and provide external validation to support the use of GCAPS as a tool for prioritization of referrals in clinical practice.

Furthermore, the absence of GCA among low-risk patients (i.e. GCAPS <9) in our cohort lends weight to the suggestion that GCAPS alone might be sufficient to exclude GCA. Of >350 patients included in the recent study by Sebastian *et al.* [5], none with GCAPS <9 ultimately had GCA. These results suggest that it might be feasible to adopt a GCAPS binary cut-off when accepting or rejecting referrals to the GCA FTP, thus avoiding imaging and/or specialist review of low-risk patients. This would help to focus resources on those truly at risk and facilitate rapid review. Our data suggest that a score of <10 (not GCA) or ≥10 (possible GCA) would be optimal for this purpose, because all GCA cases were captured (i.e. 100% sensitivity), but with fewer false positives than GCAPS <9 (i.e. higher specificity). Early results from an ongoing prospective multicentre study, presented at the EULAR Congress 2021, also indicated an optimal cut-off point ≥10 (100% sensitivity, 69% specificity) [16], and authors involved in a GCA FTP in Luton have suggested applying this value when accepting referrals [7].

A GCAPS cut-off of ≥13 was optimal in our cohort in terms of maximum combined sensitivity and specificity (93.2% and 90.6%, respectively) and maximum accuracy (91.5%), meaning the greatest number of correct final GCA diagnoses. Although less appropriate for screening referrals (where maximizing sensitivity helps to avoid missed diagnoses), GCAPS ≥13 might be a pragmatic threshold for initiation of empirical glucocorticoid treatment, with rapid GCA FTP review before initiation of glucocorticoid for GCAPS 10–12. Such an approach would minimize over-treatment, increase confidence in interpretation of negative clinical and US findings, and carry a low risk of complications.

Compared with the recent study by Sebastian *et al.* [5], a higher proportion of our patients had a final diagnosis of GCA (34% *vs* 25%), and our distribution across GCAPS risk groups was shifted to the higher end (32% low risk, 31% medium risk, 37% high risk in our study, *vs* 43% low risk, 39% medium risk, 18% high risk in the previous study). These discrepancies might reflect a more stringent approach to screening referrals to the GCA FTP in our centre, with more being rejected based on clinician judgement at the pre-assessment stage.

We found several individual GCAPS components associated with a final diagnosis of GCA, including age, CRP, constitutional symptoms (>1), ischaemic symptoms, temporal artery tenderness and temporal artery thickening. Cranial pain was non-significant, because most GCA FTP patients exhibited this symptom, meaning similar representation in GCA and non-GCA groups. Female sex was associated with lower likelihood of GCA, despite a higher GCAPS value. Although more females than males ultimately had GCA in our cohort, a higher proportion of females had non-GCA final diagnoses. These findings are broadly consistent with a recent systematic review of the diagnostic accuracy of clinical features in GCA, in which ischaemic symptoms, abnormalities of the temporal artery and high ESR were associated with GCA, whereas younger age (<70 years) and normal CRP were associated with absence of GCA; interestingly, sex was not found to affect GCA likelihood in that study [17]. In our cohort, rarer manifestations, such a visual abnormalities, extracranial vascular abnormalities, loss of temporal artery pulse and cranial nerve palsies, were not associated with GCA, probably because of low event rates and/or because these events provoked referral for exclusion of GCA, with some being found to have alternative causes. Our regression analyses were limited by sample size (which also explains the broad confidence intervals) but are, nonetheless, of interest to those involved in clinical assessment.

The strengths of our study include that GCAPS assessment was contemporaneous and prospectively documented, reducing the risk of recording bias inherent in retrospective analyses. Additionally, our GCA/non-GCA final diagnoses were supported by objective evidence in almost all cases, were confirmed at 6 months and were unaffected by missing data or loss to follow-up. We are therefore confident in the internal validity of our main exposure and outcome variables.

All patients underwent USS in our study, which is a further strength, in permitting a full assessment of USS performance in relationship to GCAPS. Overall, USS performed excellently as a diagnostic tool for GCA, consistent with its established role in clinical practice [12, 18]. We included facial, carotid and subclavian artery scanning as routine, but did not identify any cases dependent on these measurements alone, suggesting that results should be applicable to centres conducting standard temporal and axillary scans (according to Dejaco *et al.* [1]). Specificity appeared high in all risk groups, suggesting that USS is useful for excluding GCA regardless of pre-test probability. Specificity values were subject to overestimation, however, because we grouped negative and inconclusive scans together in these analyses [19]. This also contributed to a lower sensitivity estimate for the medium-risk group (33%), which had the most inconclusive scans. Nonetheless, sensitivity increased steeply across risk groups; indeed, the positive diagnostic yield in the low-risk group was nil, because the only positive USS was a false positive that led to further tests, including TAB and PET-CT (albeit this was an unusual case with possible overlapping final diagnoses). This strengthens our argument against routine USS examination for low-risk patients.

TAB was useful in selected cases to confirm or exclude GCA, particularly where USS was inconclusive. Several TABs performed after clear positive or negative USS appeared diagnostically superfluous. This is likely to reflect the natural learning curve associated with a newly established FTP (including use of TAB as a reference test in the early stages) and increasing confidence in USS over time.

Limitations largely reflect the real-world setting. Temporal arteries were assessed with a 13 MHz probe in a proportion of patients, which is below the recently recommended frequency of ≥15 MHz and is associated with reduced resolution of the US image. IMT cut-offs used routinely in our centre are adapted from published data [11, 12], rounded up for smaller vessels to account for probe availability. Although this could increase false-negative rates, diagnoses were not dependent on USS alone, and no missed diagnoses were identified at follow-up. The median time to assessment was 3 days, and the majority of patients were already treated with glucocorticoids, which is known to reduce the diagnostic value of USS [20] and might also explain our inconclusive scan rate. USS assessors were not blinded to GCAPS, meaning that prior knowledge could have influenced USS interpretation and GCA diagnosis. However, conferring pre-test probability is part of the function of GCAPS, and USS interpretation was guided by international standards. A small number of GCA diagnoses were not supported by positive USS or TAB, but we felt that a pragmatic definition that incorporated convincing clinical cases was appropriate for the type of study. Our results came from a single centre but enhance the applicability of GCAPS overall by demonstrating effective performance in a newly established GCA FTP, distinct from the original specialist unit.

Introduction of GCAPS to GCA FTP referral pathways might help to enhance the quality and appropriateness of referrals from primary care [21]. This would be likely to require training for referrers and capacity for dialogue with specialists to mitigate the possibility of inadequate assessment and underestimation of risk. In NHS Lanarkshire, we have conducted an educational meeting for general practitioners to introduce the concept of GCAPS. Although some GCAPS components are unambiguous, others (e.g. jaw claudication, temporal artery assessment, alternative diagnoses) might be subject to misinterpretation. In our experience, uncertainty tends toward over-scoring and unnecessary referral and treatment, rather than missed diagnoses. Additional validation of GCAPS performance when used in this setting would be desirable.

In summary, GCAPS performed excellently in stratifying patients by risk, and discriminating GCA from non-GCA final diagnoses. Our results provide external validation of existing data from Southend. GCAPS appears sufficient to exclude GCA in low-risk cases and might therefore have clinical relevance beyond pre-test probability (i.e. as a diagnostic tool). In future, use of a GCAPS binary cut-off as an entry requirement to the GCA FTP might reduce the need for imaging with or without specialist review in a proportion of patients.

*Funding:* No specific funding was received from any bodies in the public, commercial or not-for-profit sectors to carry out the work described in this article.

*Disclosure statement:* K.D. has received speaker fees from Menarini Pharma UK. A.C. has received speaker, travel and registration fee sponsorship from Novartis, Abbvie, Chugai Pharma, Colgene, Roche and Lilly. The remaining authors have declared no conflicts of interest.

## Data availability statement

Data underlying this article will be shared on reasonable request to the corresponding author.

## Supplementary data

Supplementary data are available at *Rheumatology Advances in Practice* online.

## Supplementary Material

rkab102_Supplementary_Data
