# Supplementary material for: Validation of the Southend giant cell arteritis probability score in a Scottish single-centre fast-track pathway
Source: Rheumatol Adv Pract. 2021 Dec 15;6(1):rkab102. doi: 10.1093/rap/rkab102 (PMC8765789; doi:10.1093/rap/rkab102)
Supplement: rkab102_Supplementary_Data [file rkab102_supplementary_data.docx]

Supplementary material

**Supplementary Table S1. Characteristics of patients with diagnosis of GCA despite non-positive additional test results.**

| **Demographic** | **ACR criteria** | **GCAPS** | **USS** | **TAB** | **Comments** |
| --- | --- | --- | --- | --- | --- |
| **Male, 64** | Fulfilled | 16 | Negative | Inconclusive | Convincing clinical picture and response to steroids; USS after 8 days and TAB after 4 weeks on steroids. |
| **Female, 69** | Fulfilled | 16 | Inconclusive | Negative | Developed scalp necrosis with medium vessel granulomatous vasculitis on skin biopsy. |
| **Male, 69** | Fulfilled | 15 | Inconclusive | Negative | Convincing clinical picture and response to steroids; USS after 2 weeks and TAB after 4 weeks on steroids. |
| **Male, 73** | Not fulfilled | 11 | Inconclusive | Negative | Initial USS done after 3 days on steroids; flared following rapid steroid taper; subsequent USS positive. |

*ACR = American College of Rheumatology. GCAPS = giant cell arteritis probability score. USS = ultrasound. TAB = temporal artery biopsy.*

**Supplementary Table S2. GCAPS additional components (i.e. not included in table 1) for all patients, by risk group, and by final diagnosis.**

|  | **All (n=129)** | **Low risk (n=41)** | **Med risk (n=40)** | **High risk (n=48)** | **GCA (n=44)** | **Not GCA (n=85)** |
| --- | --- | --- | --- | --- | --- | --- |
| **Onset (weeks):** |  |  |  |  |  |  |
| **- <6 (n, %)** | 88 (68.2) | 24 (58.5) | 28 (70.0) | 36 (75.0) | 32 (72.7) | 56 (65.9) |
| **- 6-12 (n, %)** | 17 (13.2) | 4 (9.8) | 5 (12.5) | 8 (16.7) | 8 (18.2) | 9 (10.6) |
| **- 12-24 (n, %)** | 13 (10.1) | 6 (14.6) | 5 (12.5) | 2 (4.2) | 2 (4.5) | 11 (12.9) |
| **- >24 (n, %)** | 11 (8.5) | 7 (17.1) | 2 (5.0) | 2 (4.2) | 2 (4.5) | 9 (10.6) |
| **Extracranial vascular signs:** |  |  |  |  |  |  |
| **- Bruits (n, %)** | 3 (2.3) | 0 (0) | 0 (0) | 3 (6.3) | 2 (4.5) | 1 (1.2) |
| **- Pulseless (n, %)** | 2 (1.6) | 0 (0) | 0 (0) | 2 (4.2) | 2 (4.5) | 0 (0) |
| **CN palsy (n, %)** | 2 (1.6) | 0 (0) | 0 (0) | 2 (4.2) | 1 (2.3) | 1 (1.2) |
| **Alternative diagnosis more likely:** |  |  |  |  |  |  |
| **- Infection (n, %)** | 17 (13.2) | 6 (14.6) | 9 (22.5) | 2 (4.2) | 2 (4.5) | 15 (17.6) |
| **- Cancer (n, %)** | 4 (3.1) | 2 (4.9) | 1 (2.5) | 1 (2.1) | 1 (2.3) | 3 (3.5) |
| **- Other SARD (n, %)** | 5 (3.9) | 5 (12.2) | 0 (0) | 0 (0) | 0 (0) | 5 (5.9) |
| **- Other head / neck pathology (n, %)** | 31 (24.0) | 21 (51.2) | 7 (17.5) | 3 (6.3) | 2 (4.5) | 29 (34.1) |

*GCAPS = Giant cell arteritis probability score. CN = cranial nerve. SARD = systemic autoimmune rheumatic disease.*

**Supplementary Table S3. Multivariable logistic regression results; associations of individual GCAPS components with final GCA diagnosis.**

| **Variable** | **Odds ratio** | **95% confidence interval** | **P value** |
| --- | --- | --- | --- |
| **Female sex** | 0.075 | 0.008 – 0.657 | 0.019 |
| **Age** | 4.586 | 1.171 – 17.954 | 0.029 |
| **Onset** | 0.702 | 0.212 – 2.322 | 0.562 |
| **CRP** | 7.449 | 1.797 – 30.876 | 0.006 |
| **Cranial pain** | 22.068 | 0.270 – 1805.881 | 0.169 |
| **Constitutional symptoms^1^:** |  |  |  |
| **- single** | 4.360 | 0.531 – 35.808 | 0.171 |
| **- combination** | 34.716 | 2.452 – 491.550 | 0.009 |
| **PMR symptoms** | 1.946 | 0.224 – 16.894 | 0.546 |
| **Ischaemic symptoms** | 29.964 | 3.426 – 262.070 | 0.002 |
| **Visual abnormality** | 0.201 | 0.001 – 27.847 | 0.524 |
| **Temporal artery abnormality^2^:** |  |  |  |
| **- tender** | 16.190 | 1.633 – 160.512 | 0.017 |
| **- thickened** | 92.975 | 4.589 – 1871.490 | 0.003 |
| **- loss of pulse^3^** | - | - | - |
| **Extracranial vascular abnormality** | 1.386 | 0.347 – 5.532 | 0.644 |
| **Cranial nerve palsy** | 20.224 | 0.037 – 11153.346 | 0.351 |

*1. Values given are for single vs none, and combination vs none. 2. Values given are for TA tenderness vs no TA changes, TA thickening vs no TA changes, and loss of TA pulse vs no TA changes. 3. Incalculable (no episodes of loss of TA pulse among patients with final non-GCA diagnoses). Statistical significance considered as two-sided p value <0.05. GCAPS = Giant cell arteritis probability score.*

**Supplementary Table S4. Diagnostic performance of ultrasound in all patients, and by GCAPS risk group.**

|  | **Sensitivity** | **Specificity** | **PPV** | **NPV** | **Accuracy** |
| --- | --- | --- | --- | --- | --- |
| **All patients** | 86.4% | 98.8% | 97.4% | 93.3% | 94.6% |
| **Low risk (GCAPS <9)** | N/A^1^ | 97.6% | N/A^1^ | 100.0% | 97.6% |
| **Medium risk (GCAPS 9-12)** | 33.3% | 100.0% | 100.0% | 94.9% | 95.0% |
| **High risk (GCAPS >12)** | 90.2% | 100.0% | 100.0% | 63.6% | 91.7% |

*US results were “positive”, “negative” or “inconclusive”, as defined in methods section. For these calculations, negative and inconclusive scans were grouped together. 1. Cannot be calculated as no true positive cases (no low-risk patients diagnosed with GCA). GCAPS = Giant cell arteritis probability score. PPV = positive predictive value. NPV = negative predictive value.*
